# Effects of a Problem-Based Learning Education Program for Occupational Health Nursing Using Smart Learning on Occupational Health Knowledge and Nursing Professionalism

**DOI:** 10.3390/healthcare12070737

**Published:** 2024-03-28

**Authors:** Kyung Jin Hong

**Affiliations:** College of Nursing, Kangwon National University, Chuncheon 24341, Republic of Korea; kjhong@kangwon.ac.kr; Tel.: +82-33-250-8883

**Keywords:** nursing students, education, occupational health, professionalism

## Abstract

This study aimed to develop a problem-based learning program for occupational health nursing using smart learning, and to report the program’s effects on occupational health knowledge and nursing professionalism. A quasi-experimental study was performed using a non-equivalent control group pre-test–post-test design. An occupational health nursing problem-based learning program using smart learning was developed, and students produced videos containing problem-solving strategies in groups. The experimental and control groups consisted of 34 and 29 participants, respectively. To collect data, an online survey was conducted before and immediately after the intervention. The experimental and control group’s mean scores for occupational health knowledge before the intervention were 5.74 and 7.41, respectively. Additionally, the mean scores for nursing professionalism were 3.45 and 3.66. After the intervention, both the knowledge on occupational health and nursing professionalism score improved to 8.26 and 3.64 in the experimental group. This study reported significant improvements after conducting the occupational health nursing education program. These results demonstrate the need to develop a nursing education program for problem-based learning utilizing smart learning. Moreover, filming videos in cooperation with students can improve the effectiveness of education by improving knowledge.

## 1. Introduction

Workers are important targets in nursing education, in terms of preventing potential health hazards caused by various harmful substances in the workplace and providing group-based health care. In 2021, a total of 122,713 compensable injuries occurred in workplaces [1]. Therefore, it is crucial for health managers to ameliorate the work environment and provide routine and special health checkups with proper follow-up to prevent and manage such injuries. As people spend a significant part of their day at the workplace, health management there is ultimately equivalent to promoting the health and well-being of the public. This further highlights the significance of the role of occupational health managers. A previous study reported that hiring an occupational health manager has financial benefits for the company, such as reduced health care cost for the employees and decreased absenteeism owing to illness [2].

According to the Occupational Safety and Health Act of South Korea, licensed or certified personnel, such as registered nurses, physicians, industrial hygienists, atmospheric environment engineers, and ergonomic engineers, are eligible to become health managers, and registered nurses account for the majority (72%) of health managers in manufacturing industry [3]. Thus, as prospective nurses, nursing students are required to understand workers as a target of their nursing care and develop the competencies required to become a health manager. Therefore, most nursing curricula include occupational health nursing in the Community Health Nursing course for fourth-year students in South Korea. The content of the training includes the aims, history and implications of occupational health nursing, and knowledge relevant to the work of health managers.

The objectives of occupational health nursing are as follows: first, maintain a high level of, and improve, workers’ physical, mental, and social well-being; second, prevent health problems caused by working conditions; third, protect workers from health hazards; and fourth, place workers in a physically and mentally fitting work environment [4]. Thus, nursing students study work environment management and the principles and details of health management for workers based on the Occupational Safety and Health Act. However, students who have never been employed may experience challenges in understanding the different work processes of various workplaces and relevant nursing processes. Additionally, there is, generally, insufficient on-site and simulation training for occupational health nursing compared to other areas of nursing. Therefore, it is difficult to conclude that students have been adequately prepared and informed regarding occupational health nursing. In this context, this study developed a problem-based learning (PBL) program for occupational health nursing, comprising presentation of actual or potential occupational health nursing scenarios and group-based problem-solving processes.

The modern education system has shifted away from the traditional approach of one-sided knowledge transfer, such as lectures and rote learning, and places greater emphasis on learner-centered, voluntary activity-based education [5]. PBL, a popular approach in this regard, involves students’ exploration of the solutions to problems based on real-world situations through discussions and collaborations within small groups [6]. This method of learning enables students to develop a sense of ownership by collecting and analyzing different concepts and information, and ultimately identifying the solution [7]. In other words, the benefits of PBL are that it facilitates self-directed learning and develops cooperative skills. As PBL has been found to be effective in boosting comprehension, interest, and satisfaction within the class and fostering a sense of responsibility and ownership compared to other educational approaches [8], there is a need to develop PBL-based programs for occupational health nursing also. In the present study, we developed and improved the fidelity of the PBL approach by providing work environment measurement equipment and personal protective equipment so that the students could use the equipment in their problem-solving process.

The developed education program integrated PBL with smart learning techniques. Smart learning is an educational approach that enables students to achieve learning outcomes through collaborative learning using smart devices, related tools, applications, and even social media platforms [9]. This teaching method increases learners’ engagement and motivation, while also bolstering their ability to retain the acquired information [10]. Given the widespread use of smart devices among college students, this approach empowers them to engage in self-directed learning as they explore and utilize information through their devices, and visually express and share the information with others during the PBL exercises. This process involves independently seeking out information, visually presenting it, and then sharing it with fellow students, fostering a dynamic and self-directed learning environment. The educational program outlined in this context incorporates smart learning techniques to encourage students to autonomously retrieve information for problem-solving purposes. Ultimately, students are tasked with producing videos using their smart devices based on the solutions they develop. This strategy facilitates effective communication among learners and between learners and educators, utilizing visual outcomes as a conduit for meaningful interactions [5].

By having students film a video as part of the curriculum, the visual–auditory aspect enhances students’ understanding of the target, while the creative process fosters critical thinking [11]. Therefore, the education curricula in various disciplines have included video or film production—for example, studies on the amelioration of biases toward people with mental disorders through film production, and studies on the changes in perspectives toward the teaching profession among prospective teachers [12,13]. Additionally, the process of video production requires team members to collaborate, which naturally cultivates their social skills [14]. Hence, filming the problem-solving process using a smart device and sharing the final video can maximize the effectiveness of PBL.

The priority objective of occupational health nursing education is to acquire a basic knowledge of occupational health. Thus, we examined whether students’ knowledge of occupational health increased after the program, and also examined the changes in their nursing professional values (NPVs). NPVs refer to a structured perspective on nursing as a profession, encompassing views on nursing activities and the role of nursing [15]. As students engage in self-directed problem-solving, they come to recognize the expertise, autonomy, leadership, and initiative involved in the nursing profession, which may, in turn, enhance their NPVs. NPVs are at the core of holistic nursing care; they promote correct values in nursing and boost students’ employment preparation behaviors [16]. This further highlights their significance for fourth-year students—they encourage engagement in clinical practice along with a positive and independent image of nurses. Therefore, this study developed a PBL education program for occupational health nursing using smart learning and evaluated its effects on occupational health knowledge and NPVs. This study aimed to investigate the effects of a PBL education program for occupational health nursing using smart learning on the occupational health knowledge and NPVs of nursing students.

## 2. Materials and Methods

### 2.1. Study Design and Sample

This is a quasi-experimental study using a non-equivalent control group pre-test–post-test design to investigate the effects of a PBL education program for occupational health nursing using smart learning on the occupational health knowledge and NPVs of nursing students. Fourth-year nursing students taking the Community Health Nursing class, which includes sections on occupational health nursing, at the K University in C city and G University in I city, South Korea, were recruited. With cooperation from the professors at each university, the students at K University were set as the experimental group completing the developed PBL education for occupational health nursing using smart learning, and the students at G University were set as the control group. There was no significant difference in the curriculum content and operating hours for occupational health nursing education between the two universities, while the control group school operated lecture-oriented classes rather than PBL. Among the students taking the course, only those who signed an informed consent form were enrolled in the study. Using the G*power 3.1.9.4 software, the minimum sample size for a *t*-test involving two independent groups with a significance level of 0.05, power of 0.80, and effect size of 0.80 was calculated to be 52, with 26 for each group. Considering potential dropouts, the pre-test was administered on 38 in the experimental group and 42 in the control group. After excluding four students in the experimental group and 13 in the control group who did not complete the post-test, a total of 34 participants in the experimental group and 29 in the control group (from a total of 63) were included in the final analysis.

### 2.2. Instruments

#### 2.2.1. Participants’ General Characteristics

Participants’ gender was asked for. Satisfaction with their major was determined using a single question rated on a scale from 1 “not satisfied at all” to 5 “very satisfied”. “Generally satisfied” and “very satisfied” responses were considered satisfied, and “neutral” or below were considered “dissatisfied”. As we measured changes in occupational health knowledge in this study, we used a yes-or-no question about having visited a workplace health management site. The need for occupational health nursing education was determined with a single question rated on a scale from 1–5 (“not needed at all” to “needed very much”), with responses categorized under two categories (≥needed in general vs. ≤neutral).

#### 2.2.2. Occupational Health Knowledge

Occupational health knowledge was assessed using a tool developed by Park [17], where respondents indicated the accuracy of 10 statements using “yes”, “no”, (or “exists”, “does not exist”), or “don’t know” options. The 10 items pertain to the Occupational Safety and Health Act, emergency response to an accident, management of hazardous substances, workplace hygiene, risks to young people and female workers, installation and operation of ventilation systems, use of personal protective equipment (PPE), regular health checks, prevention of occupational diseases, and qualifications of health managers. One point was given for each correct answer, and 0 for each incorrect answer; a higher score indicates a higher level of occupational health knowledge. The Kuder–Richardson Formula 20 (KR_20_) was 0.35 in the pre-test, and 0.37 in the post-test.

#### 2.2.3. Nursing Professional Values

NPVs were measured using the tool developed by Yeun et al. [18]. This instrument has 29 items and consists of five domains rated on a scale of 1–5: self-concept of the profession (nine items), social awareness (eight items), nursing professionalism (five items), the role of the nursing service (four items), and nursing originality (three items). Negatively worded items were reverse coded, with a higher score indicating higher NPVs. The reliability (Cronbach’s α) of the instrument was 0.92 in the previous study, whereas in the current study it was 0.93 in the pre-test and 0.94 in the post-test.

### 2.3. Study Procedure

#### 2.3.1. Development and Implementation of Occupational Health Nursing Education Program

First, we developed a “PBL education program for occupational health nursing using smart learning” for nursing students. The primary developer of the program was the first author of this study, who has a nurse license and industrial hygienist certification, with rich experience in occupational health inspections and teaching.

The education program was designed as a five-session program over three weeks, for a total of six hours. Table 1 presents the detailed contents, method, and duration of each session. Session 1 was an asynchronous online session using a pre-recorded lecture. The one-hour lecture covered topics such as the history of occupational nursing, occupational health personnel and the status of occupational injuries. Session 2 was a two hour face-to-face lecture on the main workplace hazards, the content and purpose of measuring the work environment, and criteria for exposure to hazards. In Session 3, students were grouped into teams of 5–6, and each group was presented with a scenario in which workers are exposed to one of six hazardous factors: noise, dust, asbestos, benzene, high temperature, and musculo-skeletal straining tasks. Each group was tasked to describe the hazardous nature of the given situation, the relevant legislation to prevent health hazards caused by the identified hazardous factor, any additional information to be collected and a proposed plan to improve the identified situation in their scenario. Students were free to use smart devices for this purpose, such as smartphones and tablet PCs. Furthermore, they created a storyboard outlining the scenario for the video, characters in the scenario based on the proposed plan, and the role of each group member, such as cameraman and editor, in order to film a video.

In Session 4, a video was filmed based on the storyboard created in Session 3. Each group was given approximately 40–50 min for the filming and editing, and the video length was capped at 3–5 min. After completing filming, the students gathered in the lecture room. The group leader briefly introduced the scenario, the associated hazard and played the video. Next, the professor provided feedback and delivered a short lecture, primarily regarding additional information to be mentioned. The video used benzene as an example, depicting education using a benzene material safety and data sheet (MSDS) and showing a worker correctly wearing a gas mask for health hazard prevention. The instructor provided additional lectures on the meaning of pictograms in the MSDS, the significance of regularly replacing PPE, and how to manage the PPE. In Session 5, a one-hour lecture was given on health hazard prevention and management that had not been covered in the PBL exercise. Table 2 shows the specific problem scenarios during the PBL exercise, equipment provided for problem-solving, and the contents of the videos filmed by the students. Using noise (a physical factor) as an example, the scenario described a worker engaged in automotive sheet metal work diagnosed with noise-induced hearing loss. The students were provided with a cumulative noise dosimeter, earplugs, and ear cover. They used these tools to create a scene simulating noise exceeding 90 dB(A) and filmed a scene where workers were educated about how to properly wear the hearing protective equipment.

#### 2.3.2. Data Collection

The pre-test was administered between 12 April and 2 May 2023, before providing the education program, and the post-test was administered between 5 June and 15 June 2023, after the completion of the program. Eligible fourth-year students of the two schools were informed about the purpose and method of the study and were provided with a link to the online survey. Only those who voluntarily consented to the study were allowed to proceed with the survey.

### 2.4. Data Analysis

The collected data were analyzed using the SPSS/WIN 26.0 software. Participants’ general characteristics and dependent variables were presented with descriptive statistics. Homogeneity of the two groups in their general characteristics and dependent variables were analyzed using the χ^2^ test, Fisher’s exact test, and independent t-test, and differences in the pre-test and post-test scores for each group were analyzed with the paired t-test. The Shapiro–Wilk test confirmed that the post-program NPVs score (dependent variable) met the assumption of normality, and the effects of the developed occupational health nursing education program were analyzed with repeated measures ANOVA. The reliability of the instrument was assessed using KR_20_ and Cronbach’s α.

### 2.5. Ethical Considerations

Data were collected after obtaining approval from the Institutional Review Board (IRB) at the first author’s affiliation (IRB No. KWNUIRB-2023-03-007-002). The IRB-approved recruitment announcement and information sheet were presented at pre-test and post-test, and only those who agreed to consent after reading these proceeded with the survey. The recruitment announcement contained the link to the survey and, if the survey was closed midway, the responses were not saved. To ensure that no disadvantages were present for study participation or responses, personally identifiable information was not collected. Students were asked to create a personal ID with immediate duplicate check, and the pre-test and post-test were paired using the ID. Students’ phone numbers for the purpose of sending the mobile coupon as compensation was collected using a separate link after the completion of the survey, such that the phone number was not matched to the survey responses. The phone numbers were discarded immediately after sending the mobile coupon.

## 3. Results

### 3.1. Homogeneity of General Characteristics and Dependent Variables

Table 3 shows the homogeneity of the two groups in terms of general characteristics and dependent variables. There were no significant differences in gender, satisfaction with major, prior visit to workplace health management site, and perceived need for occupational health nursing education between the two groups. The occupational health knowledge score (dependent variable) was significantly higher in the control group (7.41) than the experimental group (5.74) (t = −4.91, *p* < 0.001). Regarding specific questions, all students correctly answered the question regarding the “workplace hygiene”; however, the correct answer rate for the question regarding “management of hazardous materials” differed most significantly between the control group (60.9%) and experimental group (23.5%) (χ^2^ = 13.09, *p* < 0.001). The correct answer rate for the questions regarding “emergency response to an accident” and “installation and operation of ventilation systems” also differed between the two groups (χ^2^ = 3.91, *p* = 0.048; χ^2^ = 9.91, *p* = 0.002). The mean NPVs score did not significantly differ between the experimental group (3.45) and control group (3.66) (t = −1.59, *p* = 0.117), and the scores for each domain did not significantly differ between the two groups.

### 3.2. Effects of the PBL Education Program for Occupational Health Nursing Using Smart Learning

Table 4 shows the results pertaining to the effects of the PBL education program for occupational health nursing using smart learning on occupational health knowledge and NPVs of nursing students. The experimental group showed a 2.53-point increase in occupational health knowledge from 5.74 to 8.26 (t = −8.37, *p* < 0.001), while the control group showed a 0.72-point increase from 7.41 to 8.14 (t = −2.31, *p* = 0.028). Both groups had significant improvements in the score at the post-test. Additionally, there was a significant group and time interaction (F = 17.07, *p* < 0.001).

The experimental group showed a significant 0.19-point increase in NPVs score from 3.45 at pre-test to 3.64 at post-test (t = −3.72, *p* = 0.001), while the control group showed an insignificant 0.03-point increase from 3.66 at pre-test to 3.69 at post-test (t = −0.25, *p* = 0.807). However, there was no significant group and time interaction effect of the PBL education program for occupational health nursing using smart learning on NPVs (F = 2.37, *p* = 0.129). By domain, the experimental group showed a 0.22-point increase in the social perception score from 2.94 at pre-test to 3.16 at post-test (t = −2.95, *p* = 0.006), while the control group had a 0.07-point decrease in the score from 3.13 at pre-test to 3.06 at post-test, resulting in the largest post-test score difference between the two groups in this domain. Regarding the nursing expertise domain, the experimental group showed a 0.24-point increase from 3.75 at pre-test to 3.99 at post-test (t = −2.83, *p* = 0.008), while the control group showed a 0.01-point decrease from 3.99 at pre-test to 3.98 at post-test (t = 0.16, *p* = 0.087). However, there was no significant group and time interaction for the two domains (F = 3.39, *p* = 0.070; F = 3.07, *p* = 0.085).

## 4. Discussion

This study was conducted to develop, implement, and evaluate the effects of a PBL education program for occupational health nursing using smart learning with fourth-year nursing students. In particular, the program was designed to enable the students to use actual equipment for work environment measurement, film a video about the improvements to the problem presented to each group, and share the video to enable student–instructor interaction using visual data.

Smart learning is an educational approach facilitating self-directed learning using familiar devices, such as smartphones and tablet PCs, among students, transforming them from passive learners to active participants [19]. Integrating PBL, which involves solving a given problem situation through cooperative and collaborative activities in the group, with smart learning provides novel stimuli to students and enables real-time communication with the instructor and team members, thereby engaging them in the class activity and enhancing their confidence [5]. In particular, we enhanced the effectiveness of the approach by incorporating a video filming activity. Throughout the video creation process, students engaged in idea generation, consensus building, concretization, expression, and sharing. This enabled them to discover new information and reap the benefits of group creativity, which in turn provides experiences of collaboration and communication, negotiation and adjustment, and understanding of diverse perspectives and insights [20].

Prior to the program, the occupational health knowledge score was different between the experimental group (5.74) and control group (7.41). The difference in the scores seems to be attributable to the differences in the percentage of students with a prior visit to an occupational health site, and subsequent studies should strive to ensure homogeneity of the two groups. Additionally, the mean score was substantially different from that (7.81) found among workers in five workplaces using the same instrument [21], suggesting that nursing students considerably lack knowledge regarding occupational health without education, compared to workers. These results highlight the need to offer occupational health education for nursing students, who may later work as occupational health managers or be exposed to hazardous work environments as nurses, and to continue to develop more effective educational approaches.

The item with the highest and lowest correct answer rates were the same as those in a previous study [21]. Of 10 items, the one with the highest correct answer rate pertained to the “provision and use of hygiene facilities”, and all students believed that the use of protective suits and taking a shower after work helps prevent work-related diseases. The reason may be attributable to the continued inclusion of aseptic techniques and hygiene for infection control in the nursing curriculum. It may also pertain to the current environment, with a heightened emphasis on personal hygiene practices and environmental management in the context of the COVID-19 pandemic [21]. The item with the lowest correct answer rate pertained to the “protection of young and female workers”. The low awareness of the vulnerability of female workers appears to be attributable to the fact that the majority of the students are single females and have accepted female employment as a natural phenomenon, as they have been exposed to and trained in the nursing profession, a predominantly female profession, through their clinical practice. Further education is required to increase the students’ awareness of the characteristics of female workers and the significance of protecting female workers.

Both the experimental and control groups showed an improvement in occupational health knowledge at post-test, but the degree of improvement was significantly higher in the experimental group (2.53) than in the control group (0.72). Additionally, the occupational health knowledge score at post-test was 0.12 points higher in the experimental group than in the control group. This result supports the conclusion that the developed education program is effective in imparting occupational health knowledge in nursing students. In the future, more workplace hazards, such as job stress and radiation, should be included in the PBL education program for occupational health nursing to further boost the effectiveness of the education.

In our study, fourth-year nursing students showed a mean NPVs score of 3.45 in the experimental group and 3.64 in the control group, which was lower than that (3.93) reported among 132 fourth-year students in a single university using the same instrument [22] and similar to that (3.55) reported among 142 current third- and fourth-year students at two universities [23]. Although our results were obtained from a few fourth-year students at two universities, and thus, cannot be directly compared to the literature, the results still suggest the need to improve the curriculum and education programs for fourth-year students in order to establish ideal values and develop nursing professionalism. As fourth-year nursing students begin actively preparing for employment, developing positive NPVs induces them to take pride in nursing, and consequently, motivates them for employment preparation, further accentuating the significance of efforts to foster NPVs in nursing students [24].

Among the domains of NPVs, the domains with the highest score were expertise in nursing and properties of nursing practice, and the domain with the lowest score was social awareness, similar to the findings obtained from 223 nursing students at a single school [25]. However, the experimental group showed a significant improvement in professional self-concept, social awareness, nursing professionalism, and the roles of nursing service after the education program, while the control group showed no significant improvements in any of the domains. Filming a video about a nurse’s role in a case that is highly probable in a real-life occupational health setting and utilizing various types of equipment during this process seems to have increased the students’ awareness of nursing expertise and perceived social position, and the significance of occupational health nurses who lead occupational health management for workers. As occupational health nursing often involves little practical experience for students, theory courses need to use various group-based approaches, such as smart learning and PBL, to expose students to broader aspects of nursing beyond clinical practice. Through such exposure, fourth-year nursing students preparing to transition to practice as nurses would be able to develop a positive and professional image of nurses and further nurture their NPVs, even as they advance their careers as nursing professionals [26].

However, there was no significant group and time interaction effect on NPVs. The scores for social perception and nursing expertise domains actually decreased in the control group but increased by 0.22 and 0.24 points, respectively, in the experimental group. These results suggest that applying educational strategies that incorporate smart learning, PBL, and video filming in other areas of nursing might help instill a professional image of nursing among students. In the future, studies should expand the occupational health nursing education program by including other hazardous factors, and also include content that allows students to explore measures to prevent other work-related diseases, such as cardiovascular and cerebrovascular diseases.

In our study, the score for the originality of nursing domain regarding NPVs actually decreased after the program. It is possible that the students had trouble reading the question properly owing to the reverse coded item or that students felt that nurses currently have limited originality as professionals. To foster awareness of nursing originality among nursing students, additional education pertaining to evidence-based practice based on critical thinking and ways to develop an interdisciplinary collaborative system as an autonomous nursing professional should be provided for fourth-year nursing students.

One strength of this study is that it implemented and evaluated the effectiveness of a PBL approach using smart learning in occupational health nursing education. However, this study has a few limitations. First, the study population comprised nursing students at only two schools; therefore, the findings have limited generalizability. Furthermore, we could not control for the differences in the curricula and class times for fourth-year students between the two schools. Given that we used a non-equivalent quasi-experimental design and did not have group homogeneity in occupational health knowledge, subsequent studies should ensure homogeneity between the two groups and re-evaluate the effectiveness of our education program. Since the difference in pre-knowledge between the two groups is large and there is a possibility that the control group had a ceiling effect, it is necessary to reconfirm the effectiveness of this educational program through further study. The differences in occupational health knowledge between the two universities could have been caused by previous experience of visiting an occupational management site or pre-study related to occupational health nursing. Moreover, the reliability of occupational health knowledge was too low. Development of a reliable instrument to measure occupational health knowledge is thus needed. Additionally, we implemented the program for all fourth-year students as a part of their formal curriculum, but only a few students participated in the survey, which leaves the risk of non-response bias.

## 5. Conclusions

This study developed, implemented, and evaluated the effects of a PBL education program for occupational health nursing using smart learning on occupational health knowledge and NPVs of fourth-year nursing students, who may choose a career in occupational health nursing and take the Community Health Nursing course. As nursing students may choose a career path in a variety of areas other than clinical settings, including occupational health, acquiring appropriate knowledge of occupational health nursing and developing a positive image of nurses based on beliefs pertaining to the nursing profession is immensely significant. In particular, for nursing students who may have never been employed, utilizing smart learning in a PBL education program for occupational health nursing using smart learning, where they engaged with real-world scenarios, used various work environment measurement equipment, and participated in discussions with peers, could enhance their understanding of the occupational health setting and offer experiential insight into nursing as a professional. Our results indicated that the developed education program improved students’ knowledge regarding occupational health and, although not significantly different from the control group, it improved their NPVs. Thus, such programs should be actively incorporated into nursing curricula.

## Figures and Tables

**Table 1:** Occupational Health Nursing Problem-based Learning Program using Smart Learning.

No	Topics	Main Contents	Teaching Tools	Duration (min)
1	Overview of occupational health (online lecture)	· History of occupational health · Major occupational health personnel · Status of occupational accidents and prevention strategies.	· PPT	60
2	Management of hazardous factors	· Major hazardous factors and health effects · Concept and methods of work environment measurement	· PPT · Quiz battle application	120
3	Problem based learning using smart learning	· Orientation · Overview of problems according to 6 hazardous factors, respectively · Discussion of management strategies to prevent the health effects that could be caused by each hazardous · Create a storyboard for video shooting of the management strategies discussed	· Problem scenarios with · key questions · Various internet sources · Textbook · Team based discussion · Occupational health · management related · equipment	60
4	Video shooting and screening	· Role assignment in each team · Video shooting using various equipment · Video screening and feedback	· Mobile phone · Occupational health · management related · equipment	90
5	Supplementary lecture	· Risk and management strategies for other hazardous factors	· PPT · Quiz battle application	30

**Table 2:** Major Topics and Key Contents of Problem-Based Learning Program using Smart Learning.

No	Hazardous Factor	Problems Situation Presented	Supplied Equipment	Key Contents Filmed in the Video
1	Noise	· Auto repair shop where noise-induced hearing loss among workers occurred.	· Noise dosimeter · Earplugs · Ear covers	· Correct method to wear earplugs and ear covers · Measuring the noise using a dosimeter
2	Dust	· Furniture factory with wood cutting process	· Dustproof masks · Air pump for sample collection (high flow) · Smoke tester to test airflow · Anemometer	· Method to wear dustproof masks · Measurement of wind speed of local exhaust system using an anemometer
3	Benzene	· Auto repair shop where leukemia occurred after performing automobile painting work using benzene-containing paint	· Gas masks · Air pump for sample collection (low flow) · Smoke tester to test airflow · Anemometer	· Method to wear gas mask · Material safety data sheet (MSDS) for benzene
4	Asbestos	· Newly employed workers preparing asbestos dismantling work	· Protective clothing · Protective masks	· Method to wear protective clothing and mask · Order of assessment for asbestos dismantling work site
5	Extreme heat exposure	· Construction site where employees work outdoors in a heat wave	· WBGT measuring instrument	· Measuring WBGT · First aid for heat stroke worker
6	Musculoskeletal disorder related work	· Factory with more than four hours of welding per day	· Musculo-skeletal hazard survey paper · OWAS assessment tool	· Assessment of work posture using OWAS tool · Stretching methods to relieve the burden on the Musculo-skeletal system

**Table 3:** Homogeneity Test for General Characteristics and Dependent Variables between Experimental and Control Groups (N = 63).

Characteristics	Categories	Exp. (n = 34) *	Cont. (n = 29) *	χ^2^ or t (*p*)
		n (%) or M ± SD	n (%) or M ± SD	
Gender	Male	6 (17.6)	5 (17.2)	<0.01 (0.966)
	Female	28 (82.4)	24 (82.8)	
Satisfaction with nursing major	Satisfied	24 (70.6)	22 (75.9)	0.22 (0.638)
Experience with visiting occupational health management field ^†^	Yes	1 (2.9)	5 (17.2)	0.086
Perceived necessity of occupational health nursing education ^†^	Yes	27 (79.4)	28 (96.6)	0.060
Knowledge of occupational health	1–10	5.74 ± 1.33	7.41 ± 1.38	−4.91 (<0.001)
The laws on industrial safety ^†^	Correct	30 (88.2)	25 (86.2)	>0.999 ^†^
First aid in case of an accident	Correct	15 (44.1)	20 (69.0)	3.91 (0.048)
Management of hazardous materials	Correct	8 (23.5)	20 (69.0)	13.09 (<0.001)
Workplace hygiene	Correct	34 (100.0)	29 (100.0)	
Risks of young people and women and labor	Correct	5 (14.7)	10 (34.5)	3.37 (0.066)
Ventilation	Correct	15 (44.1)	24 (82.8)	9.91 (0.002)
Use of personal protective equipment	Correct	13 (38.2)	15 (51.7)	1.15 (0.283)
Regular health screenings	Correct	30 (88.2)	26 (89.7)	0.03 (0.858)
Occupational disease prevention	Correct	30 (88.2)	28 (96.6)	1.48 (0.224)
Health manager qualifications	Correct	15 (44.1)	18 (62.1)	2.02 (0.155)
Nursing professionalism	1–5	3.45 ± 0.40	3.66 ± 0.64	−1.59 (0.117)
Self-concept of the profession	1–5	3.70 ± 0.49	3.89 ± 0.56	−1.46 (0.151)
Social awareness	1–5	2.94 ± 0.50	3.13 ± 0.95	−0.98 (0.335)
Professionalism of nursing	1–5	3.75 ± 0.57	3.99 ± 0.65	−1.56 (0.124)
The roles of nursing service	1–5	3.75 ± 0.43	3.94 ± 0.72	−1.24 (0.221)
Originality of nursing	1–5	3.19 ± 0.73	3.47 ± 0.68	−1.59 (0.118)

* Exp. = experimental group, Cont. = control group. ^†^ Fisher’s exact test.

**Table 4:** Effects of the Occupational Health Nursing Problem-Based Learning Program Using Smart Learning on Occupational Health Knowledge and Nursing Professionalism (N = 63).

Variables	Group	Pre-Test		Post-Test		Difference (Paired *t*-Test)		Source	F (*p*) *
		Mean ± SD	t (*p*)	Mean ± SD	t (*p*)	Mean ± SD	t (*p*)		
Knowledge of occupational health	Exp.	5.74 ± 1.33	−4.91 (<0.001)	8.26 ± 1.54	0.37 (0.715)	2.53 ± 1.76	−8.37 (<0.001)	Group	8.54 (0.005)
	Cont.	7.41 ± 1.38		8.14 ± 1.13		0.72 ± 1.69	−2.31 (0.028)	Time	55.45 (<0.001)
								Group × Time	17.07 (<0.001)
Nursing professionalism	Exp.	3.45 ± 0.40	−1.59 (0.117)	3.64 ± 0.44	−0.29 (0.772)	0.19 ± 0.30	−3.72 (0.001)	Group	1.02 (0.317)
	Cont.	3.66 ± 0.64		3.69 ± 0.66		0.03 ± 0.55	−0.25 (0.807)	Time	3.98 (0.050)
								Group × Time	2.37 (0.129)
Self-concept of the profession	Exp.	3.70 ± 0.49	−1.46 (0.151)	3.93 ± 0.54	−0.29 (0.775)	0.24 ± 0.40	−3.45 (0.002)	Group	0.76 (0.388)
	Cont.	3.89 ± 0.56		3.98 ± 0.81		0.09 ± 0.62	−0.80 (0.433)	Time	6.36 (0.014)
								Group × Time	1.22 (0.274)
Social awareness	Exp.	2.94 ± 0.50	−0.98 (0.335)	3.16 ± 0.54	0.60 (0.549)	0.22 ± 0.44	−2.95 (0.006)	Group	0.09 (0.765)
	Cont.	3.13 ± 0.95		3.06 ± 0.74		−0.07 ± 0.79	0.47 (0.640)	Time	0.93 (0.338)
								Group × Time	3.39 (0.070)
Professionalism of nursing	Exp.	3.75 ± 0.57	−1.56 (0.124)	3.99 ± 0.49	1.10 (0.920)	0.24 ± 0.50	−2.83 (0.008)	Group	0.74 (0.394)
	Cont.	3.99 ± 0.65		3.98 ± 0.67		−0.01 ± 0.66	0.16 (0.087)	Time	2.44 (0.123)
								Group × Time	3.07 (0.085)
The roles of nursing service	Exp.	3.75 ± 0.43	−1.24 (0.221)	3.99 ± 0.58	−0.92 (0.359)	0.24 ± 0.43	−3.27 (0.003)	Group	1.59 (0.212)
	Cont.	3.94 ± 0.72		4.15 ± 0.74		0.21 ± 0.78	−1.42 (0.165)	Time	8.27 (0.006)
								Group × Time	0.05 (0.820)
Originality of nursing	Exp.	3.19 ± 0.73	−1.59 (0.118)	3.03 ± 0.72	−1.90 (0.062)	−0.16 ± 0.87	1.05 (0.303)	Group	4.46 (0.039)
	Cont.	3.47 ± 0.68		3.37 ± 0.69		−0.10 ± 0.70	0.80 (0.430)	Time	1.67 (0.201)
								Group × Time	0.07 (0.792)

* Exp. = experimental group, Cont. = control group.

## Data Availability

The data presented in this study are available upon request from the corresponding author.

## References

[1] Ministry of Employment and Labor (KR) Occupational Accident Statistics. Sejong: Ministry of Employment and Labor (KR). https://kosis.kr/statHtml/statHtml.do?orgId=118&tblId=DT_11806_N000&checkFlag=N.

[2] Jung M.H., Jung H.S., Lee B.I. (2013). Effect of workplace health manager’s role performance on presenteeism in the workers. Korean J. Occup. Health Nurs..

[3] Ministry of the Interior and Safety (KR) Health Manager Appointment Status. Sejong: Ministry of the Interior and Safety (KR). https://www.data.go.kr/data/15036535/fileData.do.

[4] International Labor Organization (Switzerland) Occupational Health. Geneva: International Labor Organization (Switzerland). https://www.ilo.org/safework/areasofwork/occupational-health/lang--en/index.htm.

[5] Oyelana O.O., Olson J., Caine V. (2022). An evolutionary concept analysis of learner-centered teaching. Nurse Educ. Today.

[6] Barrows H.S. (1986). A taxonomy of problem-based learning methods. Med. Educ..

[7] Sharma S., Saragih I.D., Tarihoran D.E.T.A.U., Chou F.-H. (2023). Outcomes of problem-based learning in nurse education: A systematic review and meta-analysis. Nurse Educ. Today.

[8] Lee E., Hannafin M.J. (2016). A design framework for enhancing engagement in student-centered learning: Own it, learn it, and share it. Educ. Technol. Res. Dev..

[9] Leem J.H., Sung E.M. (2015). Smart education leading teachers’ perception on characteristics of smart devices and educational possibility of smart education. J. Educ. Inf. Media.

[10] Rha I.J., Sung E.M. (2007). The effect of picture relevancy on text understanding and learner satisfaction in web-based instruction. Asian J. Educ..

[11] Multisilta J. (2014). Editorial on mobile and panoramic video in education. Educ. Inf. Technol..

[12] Kim M.G. (2019). A qualitative case study on anti-stigma efforts using independent film making. Korean J. Soc. Welf. Stud..

[13] Han E.J. (2015). Study on the change of the pre-service teachers’ viewpoint about school teachers through a smart-phone film-making instruction method. J. Korean Teach. Educ..

[14] Rasi P.M., Poikela S. (2016). A review of video triggers and video production in higher education and continuing education PBL settings. Interdiscip. J. Probl. Based Learn..

[15] Weis D., Schank M.J. (2000). An instrument to measure professional nursing values. J. Nurs. Scholarsh..

[16] Ahn S.S., Park I.S. (2022). A study on the effects of nursing professionalism and occupational values on the job preparation behavior of nursing students. J. Employ. Career.

[17] Park C.Y., Lee K.S., Lee W.C., Lee S.H. (1994). The factors associated with knowledge, attitude and practice regarding occupational health among small and medium scale industry workers. Korean J. Occup. Environ. Med..

[18] Yeun E.J., Kwon Y.M., Ahn O.H. (2005). Development of a nursing professional values scale. J. Korean Acad. Nurs..

[19] Whalley B., France D., Park J., Mauchline A., Welsh K. (2020). Developing active personal learning environments on smart mobile devices. Advances in Intelligent Systems and Computing, Proceedings of the Future Technologies Conference (FTC) 2019, San Francisco, CA, USA, 24–25 October 2019.

[20] Speed C.J., Lucarelli G.A., Macaulay J.O. (2018). Student Produced Videos-An Innovative and Creative Approach to Assessment. Int. J. High. Edu..

[21] Kim S.M., Jung H.S., Lee H.J., Lee H.Y. (2022). The effect of workers’ occupational health knowledge on the perception of job importance of occupational health manager-in the medium sized manufacturing enterprises. Korean J. Occup. Health.

[22] Kim G.Y. (2022). The convergence influence of clinical performance ability, clinical practice satisfaction, and emotional intelligence on nursing professionalism of nursing students. J. Converg. Inf. Technol..

[23] Noh G.O., Kim M.S. (2018). The influence of nursing professionalism and academic emotional regulation on college life adjustment in nursing college students. J. Korean Acad. Soc. Nurs. Educ..

[24] Park H.J., Oh J.W. (2014). The Relationships of the Clinical Practice Stress and the Major Satisfaction with the Nursing Professionalism of Nursing College Students. J. Digit. Converg..

[25] Gu H.J., Lee O.S. (2015). The Correlation between Nurse’s image, Biomedical ethics and Professionalism in Nursing Students. J. Korean Acad. Ind. Coop. Soc..

[26] Ko Y.J., Kim I.K. (2011). The relationship between professional nursing values and career preparation behaviors of nursing students. J. Korean Acad. Soc. Nurs. Educ..

